# Nasal endoscopic and CT scan alterations of the paranasal sinuses as predictors of severity in patients with cystic fibrosis

**DOI:** 10.5935/1808-8694.20130086

**Published:** 2015-10-08

**Authors:** Marcos Rabelo de Freitas, Déborah Nogueira Vasconcelos, Ângela Elizabeth de Holanda Araújo Freitas, José Holanda Maia Filho, Claudia de Castro e Silva

**Affiliations:** a Post-Doctor (Assistant Professor of Otorhinolaryngology - Medical School of the Federal University of Ceará.; b Md. Otorhinolaryngologist.; c Md. Assistant Physician - Department of Pediatric Pneumology - Albert Sabin Children Hospital.; d Md. Assistant Physician - Department of Radiology - Albert Sabin Children Hospital.; e Phd. Associate Professor of Pneumology - Medical School of the Federal University of Ceará. Assistant Physician - Department of Radiology - Albert Sabin Children Hospital. Medical School - Federal University of Ceará and Albert Sabin Children Hospital.

**Keywords:** cystic fibrosis, severity of illness index, sinusitis

## Abstract

Cystic Fibrosis (CF) results from mutation in the transmembrane conductance regulator gene, responsible for controlling secretory processes. The upper airways (UA) are usually involved in the form of chronic pansinusitis.

**Objective:**

To evaluate UA changes in patients with CF and to establish the correlations between sinonasal CT and endoscopic endonasal findings and disease severity.

**Method:**

Cross-sectional and prospective study with 20 patients older than 5 years with CF, assessing the Shwachman-Kulczycki (S-K) score, paranasal sinus tomography (CT) (Lund-Mackay score) and nasal endoscopy (Meltzer score).

**Results:**

CT scan alterations were observed in 94% of cases. Endoscopic alterations findings in the upper airways were found in 10 patients. Nasal polyps were found in 3 patients (15%). There was a correlation between the intensity of changes on the CT and S-K score (*p* = 0.0097), and between endoscopic findings and S-K score (*p* = 0.0318). There was a positive correlation between the presence of chronic colonization and endoscopic findings (*p* = 0.0325), which was not observed on the CT (*p* = 0.2941).

**Conclusion:**

There is an inverse correlation between the S-K clinical score and nasal endoscopy and CT findings. Therefore, patients who are clinically more severe according to the S-K score have greater UA involvement.

## INTRODUCTION

Cystic fibrosis (CF) is the most common fatal inherited disease among caucasians^1^. There are approximately 60,000 people with CF worldwide with an estimated incidence of 1:2906 live births among whites and 1:10338 among nonwhites in the United States. Specifically, CF incidence in African-Americans and Hispanics is 1:9200 and 1:15000 live births, respectively^2^. In Brazil, there are still many difficulties in confirming the diagnosis of this condition.

This is an autosomal recessive disorder resulting from mutations in the transmembrane conductance regulator gene located on chromosome 7, responsible for controlling secretory processes^3^. The genetic defect is carried by about one in 25 people in the population and it is expressed when a child gets the defective gene from both parents^4^. The ΔF 508 mutation is the most common and accounts for approximately 66% of the mutations around the world^2^.

This is a multi-systemic disease, characterized by a wide spectrum of manifestations and complications resulting from generalized dysfunction of the exocrine glands. In the lungs and digestive tract these glands produce thick and viscous secretions as a result of defective chloride ion transport through membranes, leading to obstruction of ducts and tissue destruction^1^.

Clinical complications include the development of chronic suppurative lung infections progressing to bronchiectasis, pancreatic insufficiency leading consequently to malabsorption syndrome (malnutrition in 85% of patients), diabetes mellitus, liver disease (especially in older patients) and urogenital dysfunction (male infertility). The sweat of CF patients is characteristically salty. The final diagnosis is established by the dosage of chlorine and sodium in sweat associated with compatible clinical manifestations^5^.

FC is still often considered as a childhood disease. The morbidity and mortality are high, but over the years, the advancement of knowledge on CF pathophysiology and treatment increased the survival of these patients^6^. Today, the median survival in the United States and Europe is approximately 37 years and over 40% of the people with CF have more than 18 years of age^7^.

The upper airways are involved in almost all patients in the form of chronic pansinusitis. CT scan is altered in almost 100% of patients with CF. However, the vast majority of children have no upper airways symptoms. The diagnosis of chronic rhinosinusitis determined only by the symptoms is underestimated because patients do not value their sinonasal manifestations^8^.

According to Umetsu et al. the sinonasal involvement could exacerbate pulmonary manifestations, since it would represent a source of infection^9^.

Nasal polyposis occurs in about 10% of children and up to 40% of adults with CF. Onset is generally at around 8-10 years of age, and it is uncommon in children under 5 years. The etiology is uncertain, but it may be associated with infection, allergy, immune factors, secretion alterations and ciliary abnormalities. The polyps are usually asymptomatic, but they can cause chronic nasal obstruction, resulting in mouth breathing, headache and smell and taste alterations^10^. The prevalence of nasal polyps is increased when the patient is examined by nasal endoscopy compared to the examination done by anterior rhinoscopy^11^.

This study attempts to correlate the severity of patients with cystic fibrosis with changes seen upon nasal endoscopic exam and CT scans of the sinuses. As for specific objectives, we expect to: investigate the most common respiratory manifestations in the upper airways of patients with cystic fibrosis; to compare endoscopic findings with CT scan findings in the paranasal sinuses; to correlate the presence of chronic pulmonary colonization with the findings of nasal endoscopy and CT scan of the paranasal sinuses; and to correlate CT findings with nasal endoscopic and clinical presentation of patients through the Shwachman-Kulczycki clinical score.

## METHOD

This is a prospective and cross-sectional study involving 20 patients with cystic fibrosis treated in the Pneumology ward of the Albert Sabin Children's Hospital (HIAS). outpatient pulmonology ward. The study was previously approved by the Ethics Committee in Research of HIAS under protocol 001/09.

We had patients between 6 and 25 years of age, of both genders, with a confirmed diagnosis of CF, evidenced by two positive chlorine and sodium tests or known CF mutations identified by genetic testing. All the children included in this study had the consent form signed by their guardians; and those of 18 years signed the term form themselves.

Patients with clinical and/or radiological signs of worsening of the lung and/or sinonasal disease at the time of the study, as well as those whose parents did not consent with their participation were taken off the study.

**Chart 1:**
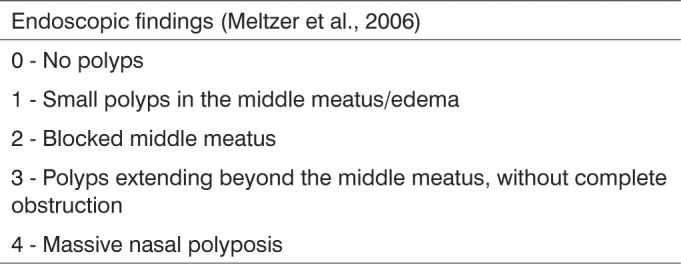
Meltzer scores used to classify nasal endoscopic findings.

The patients were evaluated by nasal endoscopy, which was performed in the outpatient otolaryngology ward of the Walter Cantídio University Hospital of the Federal University of Ceará (HUWC-UFC) using the 3.4 mm flexible optical scope from Olympus BF type 3C40 (Olympus Corporation, Tokyo, Japan) or the rigid scope of 0° with 4 mm in diameter, from ASAP (Asap Endoscopic Gmgh Products, Freiburg, Germany). Prior to starting the exam, we sprayed the subject's nose with a naphazoline-hydrochloride 0.05% with 2% tretracaine anesthetic agent, in order to facilitate the insertion of the optical fiber and reduce patient discomfort. Through this examination we made a direct evaluation of the nasal cavity, as a means to identify the presence of anatomical abnormalities in the nasal septum and/or in the structures of the lateral nasal wall, especially looking for nasal polyps. Nasal endoscopy findings were graded according to Meltzer's criteria^12^ (Chart 1).

**Chart 2:**
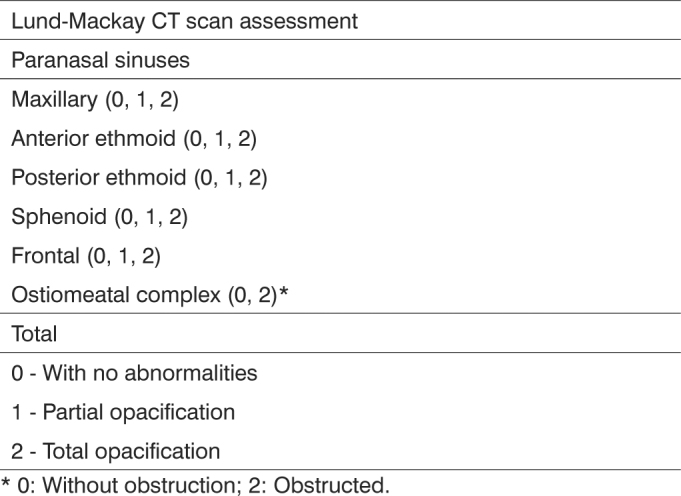
Lund-Mackay scores used to classify paranasal sinuses CT scan findings.

Was also ordered CT scans of the paranasal sinuses of 2.0 to 3.0 mm thick slices in the coronal and axial planes - low-dose technique (130 KV and 40 mA) - from the radiology department of Albert Sabin Children's Hospital - within 30 days of the nasal endoscopy. There was no need for sedation in these patients. The CT findings were classified according to the Lund-Mackay^13^ (Chart 2) criteria. Only pneumatized sinuses were scored. To enable result comparison, we corrected the original score in 0-24. Therefore, we multiplied the obtained value by 24 (total number of pneumatized paranasal sinuses)^8^.

**Chart 3:**
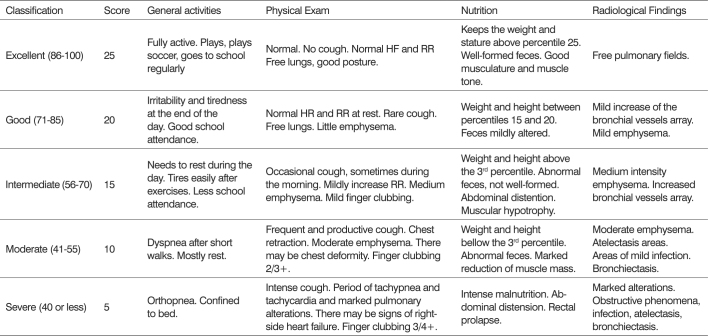
Shwachman-Kulczycki score used to classify the clinical severity of CF.

The clinical evaluation used to establish the severity of cystic fibrosis manifestations in these patients was performed using the Shwachman-Kulczycki^14^ (Chart 3) clinical score.

To process the data, create the graphs and assist with the statistical analysis we used the GraphPad Prism version 4.00. The Spearman test was used to study the correlations between the CT and nasal endoscopy findings and the Shwachman score and CT/nasal endoscopy findings. The Fisher exact test was used to study the correlation between chronic lung colonization and CT/nasal endoscopic findings. A *p* value > 0.05 was considered statistically significant.

## RESULTS

**Figure 1:**
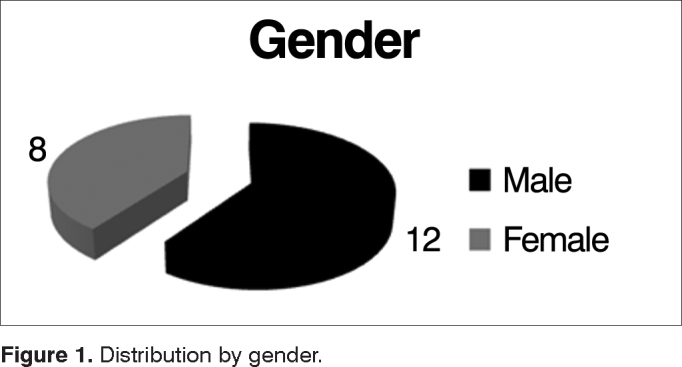
Distribution by gender.

Of the 20 patients selected to participate in the study, 8 (40%) were females and 12 (60%) were males, and the age ranged from 6 to 25 years (mean 12.6 ± 5.73). (Figure 1).

**Figure 2:**
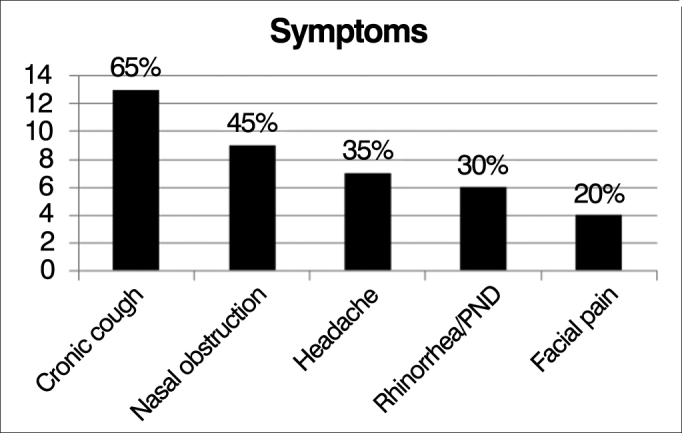
Distribution of frequencies of respiratory symptoms.

The most common airway manifestations in our patients were: chronic cough (65%), nasal obstruction (45%), headache (35%), rhinorrhea/postnasal discharge (30%), facial pain (20%) (Figure 2).

**Figure 3:**
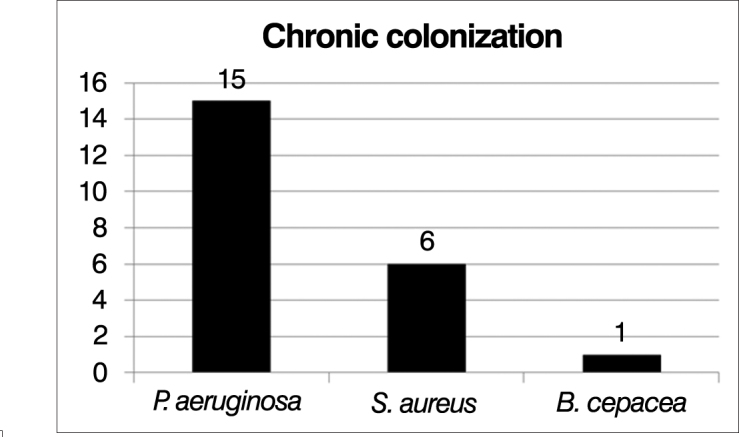
Representation of chronic lung colonization.

Of the 20 patients selected, 16 were evaluated using bronchoalveolar lavage culture to determine the presence of chronic lung colonization. Of these, 15 patients (75%) were chronically colonized with *Pseudomonas aeruginosa*; and among these, 6 (30%) were also colonized by *Staphylococcus aureus* and 1 (5%) also had *Burkholderia cepacia*. (Figure 3)

**Figure 4:**
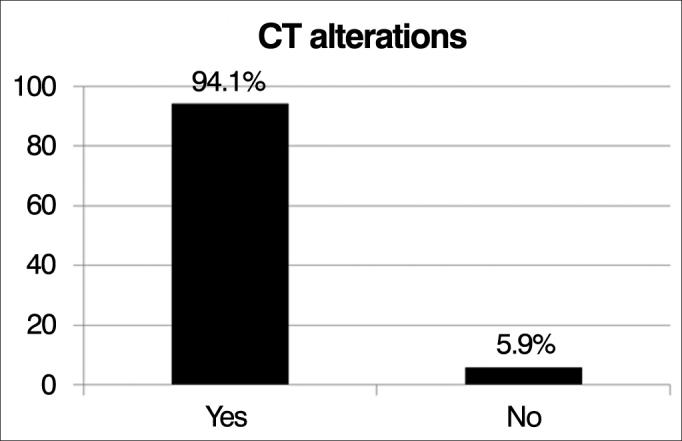
Frequency of changes seen upon computed tomography of the paranasal sinuses.

Paranasal sinuses CT scan alterations, according to the Lund-Mackay criteria, were observed in 94.1% of the 17 patients assessed (3 patients did not undergo CT: two died before the exam due to exacerbation of their lung condition and one did not come to the appointment), while only 5.9% had a normal CT scan of the paranasal sinuses (Figure 4).

**Figure 5:**
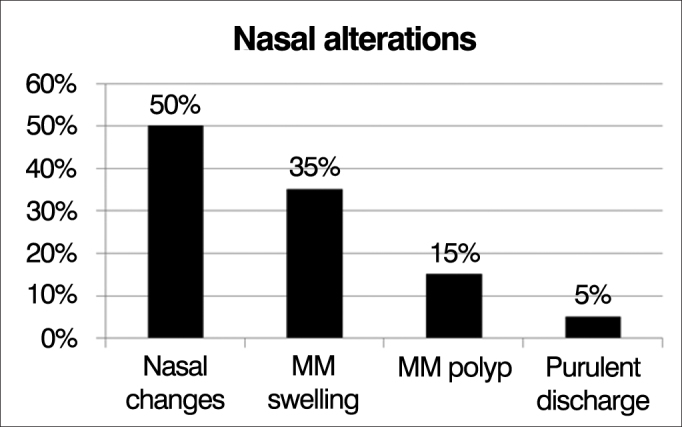
Frequency distribution of observed changes in nasal endoscopy.

Among the patients evaluated, 10 (50%) had alterations seen upon nasal endoscopic examination according to the Meltzer classification, and the most common changes were: swelling of the middle meatus in 7 (35%) patients, polyps in the middle meatus in 3 (15%) and purulent discharge from the middle meatus in 1 (5%) patient (Figure 5).

There was no statistically significant correlation between the intensity of the nasal endoscopy and sinus CT findings of in the evaluated patients (*p* = 0.4901). However, we found an inverse correlation between the clinical Shwachman-Kulczycki score and the CT imaging (*p* = 0.0097) and nasal endoscopy (*p* = 0.0318) findings, meaning that the clinically more severely ill patients had further changes in nasal endoscopy and sinus CT. (Tables 1 and 2).

**Table 1:** Shwachman-Kulczycki, Meltzer and Lund-Mackay scores adjusted for each patient.

Age (years)	Gender	Shwachman-Kulczycki	Meltzer	Corrected Lund-Mackay
6	M	50	2	24
6	F	50	2	28.8
6	F	55	2	19.2
6	F	95	2	14.4
8	M	95	0	4
8	F	80	1	38.4
9	M	60	0	28.8
10	M	40	0	7.2
10	M	60	0	NR
11	M	55	1	36
11	M	95	0	0
12	M	55	1	26.4
13	M	85	0	4.8
13	F	35	2	34
15	F	60	0	NR
16	M	60	1	36
21	F	40	2	NR
21	F	100	0	10
22	M	100	0	2
25	M	60	0	20

NR: Not performed; M: Male; F: Female.

**Table 2:** Analysis of the correlation between the Shwachman-Kulczycki (S-K) clinical score and tomographic and nasal endoscopic alterations, and correlation between nasal endoscopy and paranasal sinuses CT findings.

Spearman correlation	*p*	CI 95%
Nasal x S-K	0.0318*	-0.7799 a -0.03584
CT x S-K	0.0097**	-0.8596 a -0.1706
CT x Nasal	0.4901	-0.02366 a 0.7990

CT: Computadorized tomography; CI: Confidence interval; Nasal: Endoscopy; S-K: Shwachman-Kulczycki.

**Figure 6:**
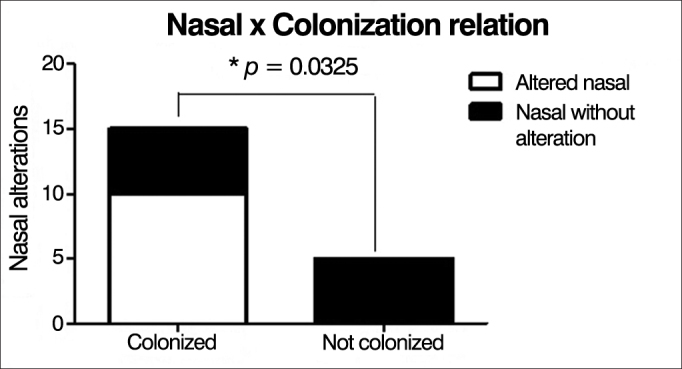
Correlation between chronic lung colonization and nasal endoscopic changes.

**Figure 7:**
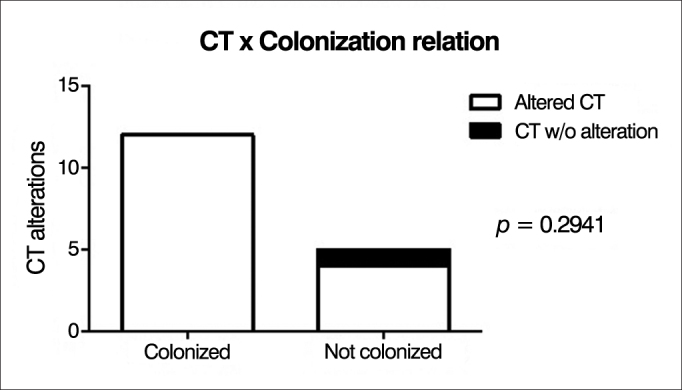
Correlation between chronic lung colonization and sinus CT changes.

We found a statistically significant correlation between the presence of chronic lung colonization and nasal endoscopy alterations (*p* = 0.0325), i.e., chronically colonized patients had higher scores on nasal endoscopy changes (Figure 6). However, this correlation was not found with changes in sinus CT (*p* = 0.2941), i.e., independent of the presence of chronic colonization, most patients had tomographic alterations (Figure 7).

## DISCUSSION

The follow up of patients with cystic fibrosis (CF) is required within the multidisciplinary approach, increasing attention from otolaryngologists, since several studies have been developed to analyze the impact of chronic rhinosinusitis on pulmonary function of patients, which can substantially increase their morbi-mortality^11^. On the other hand, treatment advances have improved the survival of these patients, allowing the inclusion of children and adults in scientific studies, such as this, addressing this condition.

The patients evaluated in our study had a high prevalence of sinonasal symptoms, such as nasal obstruction, headache, rhinorrhea/postnasal discharge and facial pain, which is in disagreement with what was described by Boari & Castro Jr^8^, who showed that the prevalence of sinonasal symptoms in CF patients is very low, explaining that this is probably due to an undervaluation of these symptoms at the expense of more severe clinical manifestations which has a higher effect on their quality of life.

Of the 20 selected patients, fifteen (75%) were chronically colonized with *Pseudomonas aeruginosa*, among these, six (30%) were also colonized by *Staphylococcus aureus* and 1 (5%) also harbored *Burkholderia cepacia*. This data is in tune with what is presented in the literature, since these pathogens are most frequently described in publications as causative agents of chronic pulmonary infection in patients with CF^3^.

The presence of changes in CT images of the paranasal sinuses according to the criteria of Lund-Mackay were observed in 94.1% of 18 patients evaluated, consistent with the descriptions of Henriksson et al. who show that virtually 100% of patients with CF also have tomographic alterations in their sinonasal area^8^, ^11^.

Ten (50%) of the patients had abnormal nasal endoscopic findings according to Meltzer's classification, matching reports from Umetsu et al^9^. showing that nasofibroscopy analysis is more sensitive to assess the presence of these findings consistent with rhinosinusitis in these patients^8^. Furthermore, still in nasal endoscopy, 15% of patients had nasal polyps, which is also consistent with the literature, describing that NP occurs in about 10% of children with CF^10^.

We found a statistically significant correlation between the presence of chronic lung colonization and nasal endoscopy changes (*p* = 0.0325); however, this correlation was not observed in paranasal sinuses CT changes (*p* = 0.2941). In the literature consulted, we did not find papers showing or corroborating this correlation.

There was no statistically significant correlation between the intensity of the nasal endoscopy findings and those from the sinus CT in the patients evaluated (*p* = 0.4901), disagreeing with what was described by Umetsu et al.^9^, who observed a statistically significant correlation between nasal endoscopy and CT findings in patients with CF. However, there was an inverse correlation between the Shwachman-Kulczycki clinical score and nasal endoscopic findings (*p* = 0.0318) and sinus CT findings (*p* = 0.0097); which means that clinically more severely ill patients had more severe changes in nasal endoscopy and sinus CT.

## CONCLUSION

The most common respiratory symptoms in the patients assessed were chronic cough, nasal obstruction, headache, rhinorrhea and facial pain.

There was no intensity correlation between the CT scan and nasal endoscopy findings.

There was a direct correlation between the presence of chronic colonization and nasal endoscopy findings, but there was no correlation with CT findings.

There was an inverse correlation between the Shwachman-Kulczyckclinical score and the tomographic and nasal endoscopic findings.
